# Empowered patient: A program to improve people with Parkinson’s communication with health care professionals

**DOI:** 10.1016/j.prdoa.2022.100156

**Published:** 2022-07-16

**Authors:** Muhammed Shahriar Zaman, Setareh Ghahari, Mary Ann McColl

**Affiliations:** a School of Rehabilitation Therapy, Queen’s University, Kingston, ON, Canada; b Department of Medicine-Division of Nephrology, Queens Univeristy, Kingston, ON, Canada

**Keywords:** Parkinson’s disease, Health communication, Program development

## Abstract

**Background:**

Communication breakdown between patients and health care professionals poses an accessibility gap preventing adequate health care. The Empowered Patient Program was developed to support people with Parkinson’s in improving their health communication skills/strategies and thus facilitate the accessibility gap in their care.

**Objective:**

Our pilot study aimed to test the feasibility and preliminary effect of the Empowered Patient Program within a small cohort of individuals with Parkinson’s disease.

**Methods:**

We completed a pre-test-post-test pilot study. Eight participants completed the Empowered Patient Program for this pilot study. Data collection was completed by administering a questionnaire prior to the program, immediately after program completion, and three months post-completion. We additionally conducted two telephone interviews with the participants to qualitatively gather feedback on the program.

**Results:**

The program elucidated statistically significant improvement across domains/areas of knowledge (p = 0.01) and self-perceived communication skills (p = 0.04) among the participants. Through feedback from the patient interviews, it was confirmed that these significant improvements were owed largely to the high level of organization, intuitive user interface, and suitable content of the program for this cohort.

**Conclusions:**

The Empowered Patient Program pilot resulted in a desired outcome indicating its satisfactory development. The next steps are to test the Empowered Patient program in a larger sample.

## 1 Introduction

For people with Parkinson’s disease (PD), routine patient-provider communication is critical because of the progressive and complex nature of the disease, the high burden of comorbidity, and the need for preventive healthcare. However, the literature highlights a concerning communication breakdown between people with PD and their health providers[1], [2]. Prior communication interventions for different patient groups have improved patient-provider communication and patient participation in health decision making^3^. Unfortunately, communication interventions for people with PD are currently underdeveloped in contrast to other clinical populations.

We have developed a communication program titled the ‘Empowered Patient Program.’ The program aims to improve knowledge about health communication, health communication-related self-efficacy, health communication and health service navigation skills in people with PD. The program consists of three sequential workshops. First, the ‘Direct Communication’ workshop educates on overcoming communication barriers by using different communication styles. Second, the ‘Difficult Health Topics’ workshop trains participants to conduct conversation on sensitive topics such as sexual health, mental health, and bowel and bladder problems. Finally, the ‘Preparing for the Appointment' workshop educates participants on healthcare professional’s (HCPs) role in the care of PD. The program is designed to be conducted in a face-to-face format for groups of six to twelve participants. The activities include group discussion, role-play, and facilitator discussion. Each workshop is 2.5 h in length.

The content of the program was developed based on an extensive literature review and expert consultation. The expert panel included people with PD, their family members, HCPs who visit people with PD on a regular basis, and the case managers of Parkinson Canada. The literature review^4^ aimed to identify specific barriers that currently limit people with PD from accessing healthcare services. A systematic search of bibliographic databases, including Medline, Embase, and CINAHL was completed for twenty-nine articles published between the years of 2000–2014 for review. The review confirmed that communication difficulties are notable barriers in accessing healthcare services for people with PD, and that low health-related self-efficacy is an essential contributor to communication breakdown. Therefore, we used the theory of Self-efficacy^5^ in developing the program. For promoting health-related self-efficacy, we designed the workshop activities to stimulate mastery and vicarious experience of patient-provider interactions. Furthermore, we included content to improve health literacy and health service navigation skills as many studies have identified these factors as further barriers in accessing healthcare.

Expert consultation was done using a qualitative study (ethical approval #6020458) to evaluate the program’s content and user-friendly interface. An expert panel was assembled from people with PD (n = 5), their family members and caregivers (n = 2), educators and case managers of Parkinson Canada (n = 4), as well as HCPs (n = 4) including physiotherapists, occupational therapists, and nurses reviewed the program manuals and provided feedback. The focus group discussion and individual interviews followed a semi-structured format guided by a series of open-ended questions (Supplementary Table 1). The expert panel concluded that the program adequately covered essential topics that could promote communication skills. They recommended changes to further adapt the program to the physical and cognitive needs of people with Parkinson’s disease (Supplementary Table 2). We refined the program using the provided expert recommendations (changes in supplementary table 2) and then conducted a pilot study. The pilot study’ (ethical approval #6021935) aimed to test the feasibility of the recruitment process, implementation of the program, and preliminary effect on health communication- related self-efficacy, knowledge, communication with HCPs and health service navigation in a small group of people with PD. This article presents the details of that pilot study.

## 2 Method:

### 2.1 Design

We completed a pilot pre-test-post-test study that collected survey data from the participants one week prior to the start of the program, immediately after the program’s completion, and three months after the program. We also completed two telephone interviews with each participant- the first interview was conducted immediately after the program completion and the second was conducted after three months. The program was conducted according to detailed, step-by-step instructions of the program manuals in an effort to enhance intervention fidelity.

### 2.2 Participants

We recruited participants using a convenience sampling method within the Toronto area through Parkinson Canada, including participants that could communicate in English who had a self-reported diagnosis of PD. No exclusion criteria were considered. We circulated study flyers through email and personal communication for potential participants to contact us if they desired to voluntarily participate in this program. Nine participants consented to take part in the program. However, one participant was unable to complete the program due to urgent medical appointments, and thus their data was excluded from the analysis. Therefore, the data of eight participants was included for the analysis. Table 1 presents the socio-demographic and clinical characteristics of the participants.

**Table 1:** Socio-demographic and clinical characteristics of the program participants (n = 8) in pre-test-post-test study.

Variables		Frequency^†^
Age		
	Median	72 years
	Min-Max	55–82 years
Sex		
	Male	3 (37.5 %)
	Female	5 (62.5 %)
Educational level		
	High School Diploma	3 (37.5 %)
	Bachelor’s Degree	3 (37.5 %)
	Post-graduate degree	2 (25.5 %)
Occupation		
	Retired	7 (87.5 %)
	Self-employed	1 (12.5 %)
Time since start of symptoms		
	Median	2.25 years
	Min-Max	1.5–24 years
Health Utility Index (score range 0 to 1)		
	Median	0.79
	Min-Max	0.18–1.00
Number of co-morbidities		
	None	2 (25.0 %)
	1–2	4 (50.0 %)
	3–4	1 (12.5 %)
	5–6	1 (12.5 %)
† Results are shown in frequency and percent, if not mentioned otherwise.		

### 2.3 Data collection

The feasibility data was collected on recruitment, retention, and delivery of the program. First, the feasibility of recruitment was determined by comparing the eligible and enrolled participants. Second, the feasibility of retention was determined by the proportion of enrolled participants who completed the program. Finally, the feasibility of program delivery was determined by administering the program evaluation component of the Health Education Impact Questionnaire (heiQ)^6^. In addition, we documented and retained field notes to record challenges that arose during program delivery.

The impact of the program was assessed through the Communication Perceived Self-efficacy Scale^7^ for communication self-efficacy, the Stanford Communication with Physician Question^8^ for communication with HCPs, the Health Service navigation component of heiQ^6^ for health service navigation, and the Communication with Healthcare Professional (CHCP) tool for knowledge of health communication. The CHCP is a nine-item questionnaire developed by the research team according to Bennett & Ritchie's guidelines^9^.

Health status and socio-demographic information were collected by Health Utilities Index 3 (HUI)^10^ and a socio-demographic questionnaire designed for this study.

The telephone interviews were semi-structured through the use of pre-set, open-ended questions (Supplementary Table 1) about program content, format, and usefulness. The mean duration of the interviews was 16.3 (±5) minutes at the post-test period and 5.5 (±2) minutes at three months.

### 2.4 Data analysis

Descriptive analysis (using SPSS v. 24) techniques were used for quantitative data that included participant's characteristics, outcome measures, and feasibility data. The Wilcoxon Signed Ranks Test was used to test for statistical significance as the data were non-parametric and met test assumptions. The missing data were treated by imputation; the item-level missing data at the pre-test period was imputed by the variable's mean; the missing data at post-test and follow-up was imputed by the Last Observation Carried Forward method. The qualitative data from interviews and field notes were analyzed using thematic analysis.

## 3 Result

### 3.1 Feasibility of the program

Feasibility of recruitment was moderate, with 8 of the fifteen (53.3 %) interested participants taking part in the program. For seven interested participants who decided not to participate, incompatible timing of the program (10:00–11:30 am) with their schedules was the reported reason.

Feasibility of retention was high, in that the percentage of participants attending all three workshops was 62.5 % (n = 5), and two workshops were 37.5 % (n = 3).

Feasibility of program delivery was also high, based on a mean score of the heiQ Program Evaluation tool of 3.85 out of 4, with the range of item-level mean scores from 3.5 to 4 (Supplementary Fig. 1). The noted engagement and demeanor of participants in the program was enthusiastic. Still, the program requires various strategies for involving participants in group discussions and role-play on difficult health topics such as sexual health, due to the nature of these often-uncomfortable topics.

### 3.2 Effect of the program

The participants improved in all administered outcome measures in the post-test and follow-up period (Table 2). However, only the improvements in knowledge and (p < 0.017) and patient’s self-perceived communication with HCPs (p < 0.041) at the post-test period were statistically significant. A 17.2 % improvement in the knowledge score and a 17.8 % improvement in the communication score were found at the time of the post-test. Descriptive analysis elucidated that younger people (below the median age of 72 years), males, and people who had low HUI scores (below the median score of 0.79) improved in knowledge and communication proportionately more than for other categories of participants. The improvement of self-efficacy (3.4 %) and health service navigation (8.7 %) was relatively low compared to improvements seen for knowledge and communication. However, it is important to note that this cohort of participants presented with a high baseline score for self-efficacy (4.13 out of 5) and health service navigation (3.11 out of 4).

**Table 2:** Preliminary effect of the Empowered Patient program in the pre-test-post-test study (n = 8).

Measure	Time point	Mean	St. Deviation	Median	Range	P-value†
Knowledge*	Pre-test	3.84	0.62	3.83	2.87–4.74	
	Post-test	4.50	0.27	4.46	4.13–5.00	0.017
*Communication Self-Efficacy*	Pre-test	4.13	0.50	4.17	3.38–4.66	
	Post-test	4.27	0.41	4.50	3.50–4.69	0.309
	Follow-up	4.23	0.24	4.34	3.81–4.44	0.674
*Communication with HCPs*	Pre-test	3.04	1.08	3.00	1.67–4.67	
	Post-test	3.58	1.05	3.67	2.33–4.67	0.041^⁑^
	Follow-up	3.46	0.92	3.67	1.67–4.67	0.348
*Health Services Navigation*	Pre-test^‖^	3.11	0.64	3.04	2.00–4.00	
	Post-test	3.15	0.32	3.10	2.60–3.60	0.833^⁑^
	Follow-up	3.38	0.23	3.40	3.00–3.60	0.357

† Level of significant was tested by Wilcoxon Signed Ranks Test; ^⁑^ The p-value is tested from pre-test to post-test;  The p-value is tested from pre-test to follow-up.

### 3.3 Feedback on the program

Participant feedback emphasized that the program's contents are relevant, meaningful, systemic and helpful for understanding health communication problems as well as for gaining skills to overcome those problems. The findings resembled the result of the heiQ program evaluation tool that showed participants (n = 8) either “strongly agreed” or “agreed” about the relevance of the program content to their own situation (mean 3.5, SD 0.53). They also said that the program changed their thought perspective from reactive to proactive and made them curious, determined, and confident about their health care. Participants expressed that they actively learned about the roles of different HCPs involved in the care of Parkinson's disease. As a participant said:

“It is sort of demystifying the family physician's role and specialist's role and everything, the difference between the two.“(Participant 2)

Overall, participants (n = 8) also said that the program's format was appropriate, encouraging, professional, and well-organized. A sample testimonial provided by one participant states:

*“I would definitely say so [format was suitable] because the discussion was wide open. In other words, it wasn't just driven one-way communication from like, say from you [facilitator] or the doctor to us. It was more information from us to each other, through the doctor and yourself. So, it was sort of round-of-the-table discussion, and I was learning from everybody. I was learning from you guys, from the other participants in the group.”*(Participant 1)

The three-month follow-up data showed that participants (n = 5) retained and used the knowledge and skills learned from the program in their subsequent healthcare visits. Participants (n = 2) further elaborated that they felt confident and empowered in communication with HCPs.

However, some participants (n = 4) reported that they felt awkward while doing the role-playing, although they believed that the activity was a good way of learning. Ultimately, they recommended displaying model role-playing, presenting case scenarios, creating an online version of the program, and considering peer-led workshops.

## 4 Discussion

The improvement of patient self-perceived health communication after attending the Empowered Patient Program was found to be greater than the change seen in self-perceived health communication skills due to other self-management programs (0.42–0.54 mean change in Empowered Patient in comparison to 0.2–0.46 in other programs)[11], [12]. This significant difference was expected and resulted from the focus of the Empowered Patient Program on varied styles and active practice of communication. In contrast, other self-management programs were developed to equip participants with self-efficacy and skills to manage participants’ overall health condition and had less emphasis on health communication. The intended content, activities, and practice sessions on health communication in the Empowered Patient Program resulted in a favourable improvement in communication skills.

The improvement in health service navigation after completing the program was not statistically significant. Different factors might have influenced the significance level of the outcome. Firstly, the sample size of the study was small. Next, participants’ characteristics, including age and duration of disease, may have influenced the outcome in ways that were not captured by this pilot study. For example, Nolte, Elsworth, Sinclair, & Osborne (2007) illustrated that younger patients are more likely to benefit from the self-management program because they present with lower baseline scores and higher motivation than older patients^13^. Similarly, patients who have been experiencing a disease for a long duration may develop health service navigation skills through their prior health care experiences. In contrast, newly diagnosed patients may not necessarily possess those same skills. Thus, older age and longer duration of disease may variably influence the outcome. Therefore, the recruitment of a heterogeneous population is recommended for a more extensive study. Furthermore, these two factors can also be controlled in the analysis of the larger study.

The pilot study recruitment was slow even though an extensive network of people with PD was used for recruitment. Furthermore, people with Parkinson’s disease who were not connected with Parkinson Canada could not be reached. Therefore, it is recommended that the larger study’s recruitment strategies target representative samples from the population, beyond those who are connected with Parkinson Canada. Additional recruitment strategies, including recruiting through hospitals, social media, print media, and community settings, can be utilized^14^. These strategies for recruiting samples have previously been found to be useful for recruiting samples for self-management studies.^14^ Furthermore, the study faced challenges in recruiting participants who lived in more remote locations because transportation is a major barrier for many people with Parkinson’s disease. Participants’ inaccessibility to the site where programs are offered is a common challenge for face-to-face self-management programs. Therefore, an alternative format of delivery, such as an online program of the Empowered Patient Program, should be considered in future. The online version of the program would also help reduce the program’s attrition rate by providing flexible completion of the workshop.

There are limitations to the study. The recruited participants were homogeneous in terms of disease severity and educational level. The mean HUI 3 (0.72) score of the study participants was comparatively higher than the HUI 3 score (95 % CI: 0.48-0.063) found in other studies ^15^, which indicated better health status of the participants. Furthermore, most of the study participants (62.5 %) had a tertiary level of education, which made them a part of the top 50 % of adult populations who have college or university level education in Canada. Literature has shown that people with higher education often have higher health literacy, thereby better health statuses^16^. Therefore, an argument can be made that the study participants were not representative of the whole of the Parkinson’s disease population who have diverse health and educational status.

## 5 Conclusion

It is anticipated that more people with Parkinson’s disease will be affected by the patient-provider communication breakdown because of growing burden of the disease. The Empowered Patient Program shows great promise in facilitating superior quality of care within existing health service systems, and amongst an increasing prevalence of PD cases.

## CRediT authorship contribution statement

**Muhammed Shahriar Zaman:** Conceptualization, Data curation, Formal analysis, Investigation, Methodology, Project administration, Resources, Validation, Software, Writing – original draft. **Setareh Ghahari:** Conceptualization, Data curation, Formal analysis, Funding acquisition, Investigation, Methodology, Project administration, Resources, Supervision, Validation, Writing – review & editing. **Mary Ann McColl:** Conceptualization, Formal analysis, Investigation, Methodology, Supervision, Validation, Writing – review & editing.

## Declaration of Competing Interest

The authors declare that they have no known competing financial interests or personal relationships that could have appeared to influence the work reported in this paper.
