# Supplementary material for: Empowered patient: A program to improve people with Parkinson’s communication with health care professionals
Source: Clin Park Relat Disord. 2022 Jul 16;7:100156. doi: 10.1016/j.prdoa.2022.100156 (PMC9310115; doi:10.1016/j.prdoa.2022.100156)
Supplement: Supplementary data 1 [file mmc1.docx]

| **Interview question for** | **Question** |
| --- | --- |
| Expert consultation | - In your opinion, how appropriate is the content of the Workshops in achieving the program purpose? - What are your thoughts regarding the format/layout of the information provided in the workshop manual in response to the needs of the population? - What are your thoughts on the length of the program? - After reviewing the workshop manual did you feel that an anything could be improved? This could include removing, adding, or changing content? |
| Pre-test-posttest study: Post-test | - After completing the program, did you find the content (the information) helpful? If so, could you please provide examples of why it was helpful? - What are your thoughts regarding the format (the way that this program was presented) of the program? Did it fit the needs of you and your community? - What are your thoughts on the duration of the program? - Is there anything you would like to add or remove from the program? |
| Pre-test-posttest study: Follow-up | - Did you see your doctors or any healthcare professionals in last three months? - Did you use any of the knowledge or skills you learned from the program during your visit to the doctors/healthcare professionals? Can you please give an example? |

Supplementary Table 1: Guiding interview questions for the expert consultation and pre-test-post-test study

| **Suggestions for program refinement by the Parkinson disease experts** | **Changes** |
| --- | --- |
| **Content of the program** | |
| Simplification of the language of the manuals | The language of the manuals was simplified to facilitate participant understanding. Use of medical jargons was avoided. |
| Addition of content in ‘Direct Communication’ and ‘Preparing for the Appointment’ workshop | The recommended content for barriers to communication, communication style, additional healthcare professionals and My Health Book were included. |
| Addition of examples on difficult health topics | No additional content on recommended topics were included to the manuals. However, instructions for the program facilitators were provided to prompt program participants to inquire about additional difficult health topics. The facilitator was instructed to include additional health topics in the discussion if participants ask to add more topics in the discussion. |
| **Format of the program** | |
| Modification of workshop activities | Ice-breaking activity: The activity of telling truths and lie was removed from the icebreaking activity. Questions on the duration of disease and participants’ hobbies were added. |
|  | Goal-setting activity: The goal-setting activity was changed to asking participants what they wish to achieve by the end of the workshop. |
|  | Difficult health topics discussion: Outline of discussion etiquette, educating participants about difficult health topics, and respecting participants’ decision of not participating in the discussion were added. |
|  | Writing activities: All writing activities were revised to allow participants to progress without writing requirements. |
|  | Stretching activities: Risky exercises were removed from the list of suggested exercises. Additional instruction was provided for completing the exercises in a seated position for inclusivity. |
| Use of additional resources in facilitator-led discussions | Use of multimedia (PowerPoint presentation, use of hardcopy handouts etc.) in the discussion was incorporated. |
| **Length of the program** | |
| Disagreement about the duration of the workshops | The duration of the workshops was kept to 2.5 hours, however, a follow-up assessment and any necessary changes to the duration will be made based on the results of the pilot study. |
| **Additional recommendation** | |
| Inclusion of caregivers in the workshops | Inclusion of caregiver in the workshops was incorporated. |

Supplementary Table 2: Refinement suggested for ‘Empowered Patient program’ by the Parkinson disease experts and the revisions

Supplementary Figure 1: Evaluation of the Empowered Patient program by participants (n=8) of the pre-test-post-test study
